# Characterization and general health conditions of workers in a Chilean industrial area: a worrying reality

**DOI:** 10.47626/1679-4435-2022-741

**Published:** 2023-02-03

**Authors:** Gabriela Paz Urrejola-Contreras, Miguel Angel Pérez-Lizama, Caterina Tiscornia-González, Alejandra Vásquez-Leiva, Daniela Pérez-Casanova, Erika Pincheira-Guzmán

**Affiliations:** 1 Escuela de Ciencias de la Salud, Universidad Viña del Mar, Viña del Mar, Valparaíso, Chile.; 2 Escuela de Nutrición y Dietética, Facultad de Medicina, Universidad Finis Terrae, Santiago, Chile.; 3 Escuela de Nutrición y Dietética, Facultad de Medicina, Universidad Andrés Bello, Viña del Mar, Valparaíso, Chile.; 4 Independiente, investigador, Viña del Mar, Valparaíso, Chile.

**Keywords:** occupational risks, occupational safety, health, industry, cardiovascular risk, riscos ocupacionais, segurança no trabalho, saúde, indústrias, risco cardiovascular

## Abstract

**Introduction:**

Within the occupational field, changes in the characteristics of work have revealed risks associated with static, repetitive work and litle physical activity, which together with individual health conditions of workers can trigger diseases and musculoskeletal disorders.

**Objectives:**

To obtain a preliminary characterization of workers in an industrial area, including their health and working conditions.

**Methods:**

This is a cross-sectional study with a quantitative approach, developed with 69 men who worked in the industrial area of Viña del Mar, Chile. A clinical and occupational evaluation was carried out with the application of the International Physical Activity Questionnaire as well as the Standardized Nordic Questionnaire.

**Results:**

The following risk factors were identified: 53.6% of the workers were smokers, 92.8% presented low levels of physical activity, and 70.3% reported feeling pain in body segments that were physically required during their work tasks. Among all workers, 63% were overweight according to their body mass index and 62% presented high systolic pressure. Pain was mostly detected in the spine, and it was slightly associated with forklif operation by older workers (t-test p < 0.05).

**Conclusions:**

Workers were in the presence of cardiovascular and occupational risks. It is necessary to promote timely education and training on health conditions and to evaluate risks associated with machinery operation in order to prevent work-related pain.

## Abstract (pt)

**Introdução:**

No âmbito ocupacional, as mudanças no estilo de trabalho têm mostrado riscos associados ao trabalho estático, repetitivo e à pouca atividade física, fatores que, somados às condições individuais de saúde dos trabalhadores, podem favorecer o aparecimento de doenças e distúrbios musculoesqueléticos.

**Objetivos:**

Obter uma caracterização preliminar dos trabalhadores da indústria, incluindo a saúde e as condições de trabalho.

**Métodos:**

Estudo transversal, com abordagem quantitativa, realizado com 69 homens que trabalham na indústria de Viña del Mar, Chile. Realizaram-se avaliação clínica e ocupacional e aplicaram-se o Questionário Internacional de Atividade Física juntamente com o Questionário Nórdico de Sintomas Osteomusculares.

**Resultados:**

Identificaram-se os seguintes fatores de risco: 53,6% dos trabalhadores era tabagista, 92,8% apresentava baixo nível de atividade física, 70,3% declarou sentir dor em segmentos corporais necessários à realização de tarefas do trabalho. Dos trabalhadores, 63% tinha sobrepeso, de acordo com o índice de massa corporal, e 62% apresentava pressão sistólica alta. As dores foram detectadas principalmente na coluna vertebral e estavam ligeiramente associadas à operação de empilhadeira por trabalhadores mais velhos (teste *t* p < 0,05).

**Conclusões:**

Os trabalhadores encontravam-se diante de riscos cardiovasculares e ocupacionais. É necessário promover educação e treinamento adequados em relação às condições de saúde e avaliar os riscos associados à operação de equipamentos, a fim de evitar dores relacionadas ao trabalho.

## INTRODUCTION

Public health has become a global concern in recent times, which is relevant considering the many changes and transformations that took place in the workplace as described by the productive force. A significant part of the tasks performed today in the industrial sector are considered static and involve less physical activity when assisted by specialized machinery, becoming monotonous, repetitive, and imposing a greater postural load on workers.^1^

Additionally, in the occupational health field, recent studies carried out with industrial workers have observed an increase in the prevalence of overweight and obesity, which are clinical conditions related to the development of chronic and musculoskeletal diseases, temporary disability, and decreased productivity.^2,3^

Eforts in occupational health have focused on articulating strategies that make it possible to visualize the health problems of workers and design policies that aim to prevent, educate, and treat those that are urgent.^4^

It is imperative that the working population under cardiovascular and occupational risk factors be evaluated and controlled through early management.^5,6^

In this context, research in occupational medicine has been emphatic in recognizing that physical inactivity and overweight in workers can be combined as prevalent factors in the generation of musculoskeletal disorders in both static and dynamic activities.^7^ In this sense, the production of musculoskeletal dysfunctions or ailments in tissues required in work-related activities has been related to the performance of physical tasks at the expense of pain, which is a symptom widely found and described by workers in the industrial area.^8^

The absence or presence of pain has been considered a parameter that allows the evaluation of occupational health, and it can be considered a factor that tends to alter the performance and productivity of workers who sufer from pain due to absenteeism and lost workdays.^9,10^

The objective of this study was to perform a preliminary characterization of the population of workers in the industrial zone of the Valparaíso region (Chile), including occupational parameters associated with work type and health-related conditions, including body mass index (BMI), cardiovascular risk, and pain.

## METHODS

This study has a cross-sectional design. It contemplated the selection of a company in the industrial area of Viña del Mar, Chile, that produces botles and distributes liquid and drink products, operating 24 hours a day. We analyzed a sample of 69 individuals who worked 8 hours a day in rotating weekly shifs: morning, afernoon, and night. Workers had one hour to rest and had lunch at the company premises.

A professional kinesiologist conducted a personal interview to obtain data on age, seniority, type of operated machinery, presence of comorbidity, smoking, and physical activity according to criteria by the World Health Organization (WHO) and the International Physical Activity Questionnaire (IPAQ), respectively. Finally, we applied a Standardized Nordic Questionnaire (SNQ) for the analysis of musculoskeletal symptoms validated for the Chilean working population.^11^

Resting systolic and diastolic blood pressure (at the end of the health survey) were assessed twice with a Bokang Bk1005 manual sphygmomanometer. The mean between the two measurements was used according to the American College of Cardiology (ACC) and American Heart Association (AHA).^12^ The following values of systolic/diastolic pressure were used: < 120/< 80 mmHg for normotensive, 120-139/80-89 mm Hg for prehypertensive, 140-159/90-99 mm Hg for hypertension grade I, 160-179/100-109 mm Hg for hypertension grade II, and 180/110 mm Hg for hypertension grade III.

Anthropometric measurements (weight and height) used a periodically calibrated scale and a height rod with 1 mm precision. Measurements were performed with the participants barefoot, wearing minimal clothing, and the mean weight of clothing was uniformly subtracted from the recorded weight.

Subsequently, the BMI (weight/height^2^) was calculated and individuals were classified using the criteria recommended by the WHO as: < 18.5 for underweight, 18.5-24.9 for normal weight, 25.0-29.9 for overweight, 30.0-34.9 for class I obesity, 35.0-39.9 for class II obesity, and > 40 for class III obesity.

In addition, a professional kinesiologist with experience in trauma applied orthopaedic tests to assess the spine. Finally, an ergonomic kinesiologist observed the tasks performed by workers in order to understand the type of work executed at the industrial site. The evaluation was then carried out by three different professional kinesiologists who are experts in ergonomic area.

### ANALYSIS AND STATISTICS

BMI and blood pressure data were shown as percentages of workers distributed in each category. The relationship between age and systolic pressure, as well as the relationship between BMI and systolic pressure, were analyzed using Pearson’s correlation coefficient (r) followed by a regression analysis postest (GraphPad). All data are expressed as means ± standard error of the mean (SEM). The comparison between systolic pressure and smoking habits was analyzed by a Student’s t-test (GraphPad). Data for positive/negative presence of pain (Milgram, Quadrant, and Lasegue) were analyzed by a Student’s t-test (GraphPad). Data regarding age vs machinery used were analyzed by a Student’s t-test (GraphPad). Results for pain in the upper/lower back and machinery used were analyzed by one-way analysis of variance (ANOVA) (GraphPad). p < 0.05 was accepted as statistically significant; NS = not significant.

### ETHICAL CONSIDERATIONS

This research was approved by the Santiago University Institutional Ethics Commitee (350/2019) according to resolution No. 012320 and the participants signed an informed consent form.

## RESULTS

Table 1 presents the characterization of workers. The average age was 34.9 years, the mean seniority in the company was 6.5 years, and the average BMI was 26.5 kg/m^2^. Regarding their habits, 53.6% were smokers and 92.8% had a sedentary lifestyle. Around 90% of the workers reported feeling body pain and 70.3% reported that pain interfered with their work activity. Most of the workers operated the forklif crane and the electric pallet truck.

**Table 1:** Characterization of workers

Characteristics	Mean ± SD	Minimum	Maximum
Seniority in the company (years)	6.55 ± 8.40	0.30	32.80
Age (years)	34.99 ± 10.96	18.00	62.00
Weight (kilograms)	78.59 ± 12.51	50.00	110.00
Height (meters)	1.72 ± 0.07	1.55	1.90
BMI (kg/m^2^)	26.54 ± 3.87	17.93	35.1 6

	n	%
Smoking habits		
Smoker*	37	53.6
Nonsmoker†	32	46.4
Physical activity		
Category I‡	64	92.8
Category II§	5	7.2
Pain		
Presence of body pain (+)||	62	89.8
Pain (+) in the body region required by work activity||	46	70.3
Machinery operator		
Electric pallet truck	30	42.0
Forklift	37	54.0
Electric stacker	2	4.0

Criteria by the World Health Organization (WHO)

* Smoked in the last 30 days. Daily consumption pattern.

† You have never smoked.

Criteria by the International Physical Activity Questionnaire (IPAQ)

‡ Low level of physical activity: you do not perform any activity or the one you do is not suficient to correspond to Category II or III of the IPAQ.

§ Moderate level of physical activity: there are three criteria to classify a person as active: a) three or more days of vigorous physical activity for at least 20 minutes per day; b) five or more days of moderate physical activity and/or walking for a least 30 minutes per day; c) five or more days of any combination of walking and/or physical activity of moderate and/or vigorous intensity, reaching an energy expenditure of at least 600 mets/min per week.

Criteria by the Standardized Nordic Questionnaire (SNQ)

|| Discomfort and/or pain present in the last seven days according to a Visual Analogue Scale (VAS).

SD = standard deviation.

**Figure 1:**
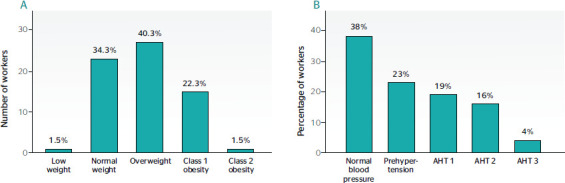
Distributions of body mass index (BMI) and blood pressure in workers. A. Number of workers vs BMI. The insert describes the percentage of workers by category. B. Distribution of blood pressure categories of workers expressed by percentage. AHT = arterial hypertension.

Figure 1A shows that 40.3% of the workers were overweight (BMI ≥ 25.0 kg/m^2^) and 1.5% presented class II obesity (BMI of 35.0-39.9 kg/m^2^).

Figure 1B indicates the distribution of participants per blood pressure category. Over 23% of the participants were considered prehypertensive and 39% were hypertensive.

When inquired whether they were being treated for hypertension, only four of them admited to being under treatment, which corresponded to 7% of the sample, while 93% were not aware that they presented hypertension.

**Figure 2:**
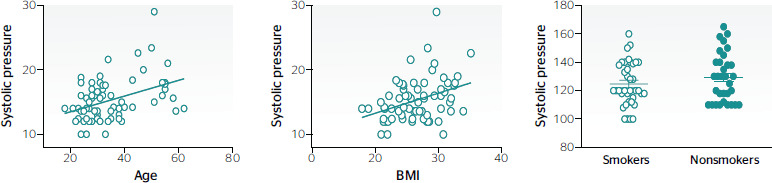
Relationships of age, smoking habits, and body mass index (BMI) with systolic pressure. A. Increase in blood pressure with increasing age. B. Increase in blood pressure with increasing BMI. C. No relationship was observed between blood pressure and smoking habits.

Figure 2 shows that higher systolic pressures are related to increases in age, but this correlation (R^2^ = 0.15) is not significant. A similar result was found for age vs BMI (R^2^ = 0.13).

A relationship with smoking was not conclusive in the case of workers with higher systolic pressure (mean systolic pressure for smokers: 124.7; nonsmokers: 129.6; t-test p < 0.05).

**Figure 3:**
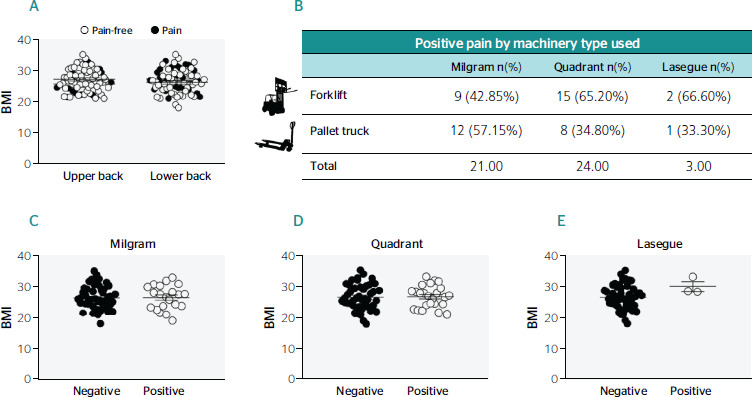
Pain, body mass index (BMI), and type of machinery used. A. Relationship between pain and BMI. There are no significant diferences between pain in the upper and lower back regarding BMI. B. Pain and type of machinery used. Positive pain results among workers who operated forklifts by orthopaedic test: Milgram (42.85%); Quadrant (65.2%); and Lasegue (66.6%). Positive pain results among workers who operated pallet trucks by orthopaedic test: Milgram (57.15%); Quadrant (34.8%); and Lasegue (33.3%). C-E. Relationships between BMI and orthopaedic tests. There were no significant diferences between BMI values regarding positive Milgram and Quadrant tests (t-test p < 0.05). However, we found significant diferences between BMI values considering a positive Lasegue test (t-test p < 0.05).

Figure 3 presents the analysis of data regarding workers who reported feeling pain: 17.4% reported upper back pain while 36.2% reported lower back pain. During the evaluation using the Milgram, Quadrant, and Lasegue orthopaedic tests, workers who operated the forklif crane presented a positive Milgram test in 42.85% of the cases, whereas 65.2% were positive for Quadrant and 66.6% for the Lasegue test. When compared to workers who operated the electric pallet truck, the same tests were positive in 57.15%, 34.8%, and 33.3% of the cases, respectively. No significant diferences were observed when relating the BMI with positive Milgram and Quadrant tests; however, the three subjects who presented positive Lasegue tests presented a significant diference (t-test p < 0.05).

**Figure 4:**
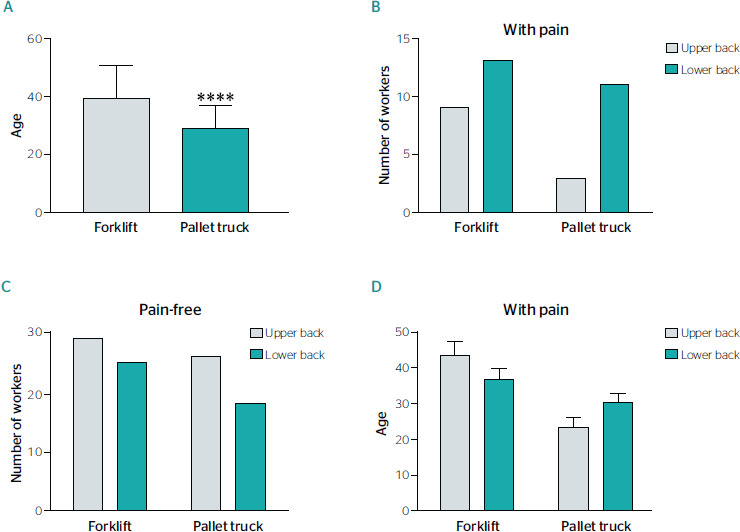
Presence of pain according to age and machinery used. A. Age and type of machinery used. Workers with a mean age of 39 years used the forklift compared to workers with a mean age of 29 years who used the pallet truck (t-test p < 0.05). B. Relationships between pain and type of machinery used. We found that pain occurred preferably in the lower back regardless of the type of machinery used. C. Pain-free individuals and type of machinery used. We did not find diferences in the number of workers without pain between the types of machinery used. D. Relationships between pain, age, and type of machinery used. We found that older workers preferentially used the forklift and felt more pain in both the lower and upper back compared to younger workers who used the pallet truck.

Figure 4 shows the analysis of pain among workers who reported pain in body regions involved in work activities, where the highest incidence was observed in the spine. Specifically, 56.7% of the workers who operated the forklif and 43.3% of those who operated the electric pallet truck reported a higher incidence of pain in the lumbar region. The average age of workers who reported pain in the lumbar region was between 30 and 35 years. On the other hand, on average 48% of the individuals were asymptomatic for pain in the upper and lower regions of the spine. The average age of pain-free participants was 40 years for forklif trucks and 30 years for electric pallet trucks.

## DISCUSSION

This study has shown that the sample of adult workers from this industrial area presents unfavorable health conditions. We can highlight the presence of cardiovascular risk factors, including elevated blood pressure in 62% of the participants, most of whom were unaware of this condition and lacked treatment (40.3%). Furthermore, 23.8% of workers were overweight and had some degree of obesity, and we obtained the alarming indicator of a sedentary lifestyle for 92.8% of the participants. These data seem to be in accordance with other Latin American studies on the working population^13,14,15^ despite interventions designed to reduce these risk conditions, which are related to the onset of chronic diseases at an early age.^16,17,18^

The workers were mostly exposed to machine-assisted tasks and roles, thus corroborating that during working hours the worker performs static and repetitive machinery control operations^19,20^ using specific body segments where there is a risk of vibration and litle physical activity that allows the recovery of musculoskeletal tissues.^21,22^

The presence of pain in 89.9% of the workers is categorical, considering also that 70.3% of them reported pain in body segments used daily for work activities. Other studies have revealed similar data, in which the presence of pain among workers was linked to postural overload, repetitiveness, use of protective equipment, and machinery operation.^23,24^

Pain was mostly reported in the lumbar spine, with suggestive signs of dysfunction expressed through positive results in the Milgram (42.8%), Quadrant (65.2%), and Lasegue (66.6%) orthopaedic maneuvers; although a relationship with the individuals’ BMI was only significant for Lasegue, the discomfort was mainly related to individuals who operated the forklif, which is similar to other studies of lumbar spine pain among workers.^25^ Studies warn about the relationship between pain in the lumbar spine and postures with a flexion patern^26^; this aspect should be considered if the forklif is operated in a siting position, as well as other criteria such as the increase in BMI and the duration of work until the end of the workday or a rest break.^27^

Among the limitations of this study, it is worth mentioning the lack of data for accessing potential metabolic syndrome in the working population, including blood cholesterol, glucose, and triglycerides. We also did not assess the postural load and repetitiveness associated with machinery operation using advanced ergonomic instruments. However, this study provides guidance on the need to deepen this evaluation in future research, leading to more precise information that allows further estimation of the health conditions of workers and associated risks in the performance of productive activities.

## CONCLUSIONS

This study shows that the working population is at risk of developing cardiovascular diseases due to high blood pressure and sufers from severe pain due to their work activities.
